# The immune system as a system of relations

**DOI:** 10.3389/fimmu.2022.984678

**Published:** 2022-09-13

**Authors:** Marc Daëron

**Affiliations:** ^1^Centre d’Immunologie de Marseille-Luminy, Aix Marseille Université-CNRS-Inserm, Marseille, France; ^2^Institut Pasteur-Université Paris Cité, Paris, France; ^3^Institut d’histoire et de philosophie des sciences et des techniques, Université Paris 1 Panthéon Sorbonne-CNRS, Paris, France

**Keywords:** physiology, immune system, nervous system, system of relations, adaptation

## Abstract

Progress in neuroimmunology established that the nervous and the immune systems are two functionally related physiological systems. Unique sensory and immune receptors enable them to control interactions of the organism with the inner and the outer worlds. Both systems undergo an experience-driven selection process during their ontogeny. They share the same mediators/neurotransmitters and use synapses for intercellular communication. They keep a memory of previous experiences. Immune cells can affect nervous cells, nervous cells can affect immune cells, and they regulate each other. I however argue that the two systems differ by three major points: 1) Unlike the nervous system, the immune system has a loose anatomical structure, in which molecular and cellular events mostly occur at random; 2) The immune system can respond to *molecules of* the *living world* whereas the nervous system can respond to *phenomena of the physical world*; 3) Responses of the immune system act both on the organism and on the stimulus that triggered the response, whereas responses of the nervous system act on the organism only. The nervous and the immune systems therefore appear as two complementary *systems of relations* that closely work together, and whose reactivities are well-suited to deal with physical and biological stimuli, respectively. Its ability both to adapt the organism to the living world and to adapt the living world to the organism endows the immune system with powerful adaptive properties that enable the organism to live in peace with itself and with other living beings, whether pathogens or commensals.

## Introduction

The nervous system has long been thought to be “immunologically privileged”, *i.e.*, ignored by the immune system. This view was challenged by the findings that “neurodegenerative diseases”, such as Alzheimer’s disease (1) and Parkinson’s disease (2) are inflammatory diseases, and that inflammatory diseases of the nervous system, such as *myasthenia gravis* (3) and, to some extent multiple sclerosis (4), have an autoimmune etiology. Antibodies against neurons (5) and acetylcholine receptors (6) were indeed identified in neurological diseases, and pathogenic autoreactive T cells in experimental autoimmune encephalomyelitis and demyelinating diseases (7). Lymphocytes (8) (9), *i.e.*, cells involved in adaptive immunity, and glial cells (10), *i.e.*, myeloid cells involved in innate immunity, were then found in normal brain. An extensive meningeal lymphatic network was described (11) (12). Neurons and immune cells were shown to share mediators and receptors, and to respond to each other’s stimulations. Finally, the nervous system (13) and the immune system (14) were found to be similarly controlled by the microbiota.

As a consequence, immunology and neurobiology became interested in each other. They exchanged concepts and vocabulary. After the notion of “immunological memory”, conceived by analogy with neurological memory (15), immunologists started talking of “immune synapses” by analogy with cell-cell contacts (16) used by neurons to communicate with other neurons and other cells, while neurobiologists talked of “neuronal group selection” by analogy with antigen-driven clonal selection of lymphocytes (17). As they became closer, neurobiology and immunology engendered “neuroimmunology” in the early 1980s. The newborn discipline was offered a *Journal of Neuroimmunology* in 1981, the first International Congress of Neuroimmunology was held in Stresa, Italy, in 1982, and an *International Society of Neuroimmunology* was created in 1987 (18). In 2020, *The Journal of Immunology* assembled a collection of review articles entitled *Neuroimmunology: To sense and protect* (19), and *Nature Reviews Immunology* launched a special series on Neuroimmunology introduced by an Editorial entitled *A Neuroimmune Odyssey* (20).

Forty years after the term was coined, neuroimmunology has become a new frontier. Changes in perspective challenged the view of the nervous system and the immune system as being two distinct biological systems. The immune system was described as a “sensory organ” that uses the same signals and receptors as the neuroendocrine system (21) and even as “the seventh sense” that informs the brain about microorganisms (22). Conversely, sensory neurons were envisioned as “critical mediators of immunity” (23) that “drive anticipatory immunity” (24). As the limits between the two systems were becoming more and more blurred, the nervous system and the immune system started to be viewed as parts of a single neuroimmune system.

The nervous system and the immune system have indeed many features in common. I will stress here that they contribute to the same general function: they enable and control the relations of the organism with the outside and the inside worlds. I will however argue that they differ by key features. Differences in their anatomy, in their cognitive repertoires and in their functional repertoires endow them with markedly different biological properties and fundamentally different biological functions. If indeed the immune system is a second system of relations, it deals with the living world whereas the central nervous system deals with the physical world. If both systems can induce the organism to respond to many different stimuli, the immune system acts on the triggering stimulus itself, whereas the central nervous system does not.

## Three types of biological systems

The *American Society of Physiology* records 10 biological systems in humans: the cardiovascular system, the digestive system, the endocrine system, the immune system, the muscular system, the nervous system, the renal system, the reproductive system, the respiratory system, and the skeletal system^1^. A biological system is an ensemble of biological objects —molecules, cells, tissues, organs, etc.— which, altogether, contribute to a given biological function. Objects belonging to a system can be similar or different, close to each other or disseminated throughout the body, linked together or not, immobile or mobile. Their only common point is the function they contribute to. Describing biological systems is one way of cutting out functional units within living organisms. These functional units depend on scientists, on their knowledge, and on what they aim at understanding. Biological systems are therefore not material objects but images that scientists project on the body to help them understand how living beings live.

If so, one is entitled to conceive other or different functional units, a neuro-immune system for example, or a neuro-endocrine system or even a neuro-immuno-endocrine system. One might also consider additional systems, a hematopoietic system and a cutaneous system for example, fulfill functions that are not fulfilled by the 10 systems listed by the *American Society of Physiology*. Besides, not all biological systems have always existed. There was no immune system before it was first proposed by immunologists in the 1960s (25). Conceived to account for the protective immunity conferred by vaccines, *i.e.*, consequences of artifactual maneuvers, the immune system became a physiological system that took place among others because it protects against pathogens. Since then, *defense* became a universal vital function, and “defense systems”, referred to as immune systems, were described in all living beings, including plants (26), protozoa (27), archaea and bacteria (28).

If one keeps in mind that, contrary to man-made artifacts, biological objects were not *made to fulfill* a specific *function*, but were selected during evolution because they conferred some critical advantage to phylogenetic ancestors (29), one can consider what biological objects do when *they function* and what *their functioning* enables. On this basis, I propose to set up biological systems into three groups: 1) *Systems of structure*, 2) *Systems of maintenance and* 3) *Systems of relations*. Systems of structure enable the *physical existence* of living beings in space and their movements. They comprise the cutaneous system, the skeletal system and the muscular system. Systems of maintenance enable the *substantial existence* —the production, the degradation and the renewal of biological objects and their substance— of living beings, their homeostasis and their reproduction. They comprise the digestive system, the respiratory system, the renal system, the endocrine system, the cardiovascular system, the hematopoietic system and the reproduction system. Systems of relations enable the *interactions* of the organism *with the world* and control them. They comprise the nervous system and the immune system.

## The immune system like the nervous system

Although not very much alike *a priori*, the nervous and the immune system share major features. Both systems perceive^2^ not only the outer world, but also the inner world, both undergo a selection process during their ontogeny, both use similar communication means, and both keep a memory of previous experiences.

### Both the nervous system and the immune system perceive the outer and the inner worlds

The nervous system perceives both the outer and the inner worlds through two types of sensory apparatus. *Unique sensory receptors* (*i.e.*, photoreceptors, hairy cell cilia, olfactory receptors, taste receptors, equilibrium receptors) located in a limited number of paired or symmetrical sensory organs located in the head (*i.e.*, eyes, outer ears, nose, tongue and inner ears) enable the perception of specific stimuli (*i.e.*, light, sounds, odors, tastes and acceleration). *Multiple sensory receptors* (*i.e.*, thermoreceptors, mechanoreceptors, baroreceptors and nociceptors) located in peripheral tissues (*e.g.*, the skin) and internal organs (*e.g.*, muscles, bones, vessels, fasciae, visceral organs), enable the perception of general stimuli (*i.e.*, temperature, touch, pressure and pain). Head and skin sensory organs receive stimuli from outside. Sensory organs present in internal tissues receive stimuli from inside.

The immune system perceives both the outer and the inner worlds through three types of immune receptors expressed by a variety of immune cells (30). Receptors of the first type are transmembrane proteins expressed at the cell surface. They include Immunoreceptors and Pattern Recognition Receptors. Immunoreceptors include T Cell Receptors (TCRs), B Cell Receptors (BCRs), Fc Receptors (FcRs) and Natural Cytotoxicity Receptors (NCRs). Pattern Recognition Receptors (PRRs) include Toll-Like Receptors (TLR1, 2, 4, 5, 6) and C-type Lectin Receptors (CLRs). Receptors of the second type are transmembrane proteins expressed at the surface of intracellular vesicles. They are the PRRs TLR3, 7, 8 and 9. Receptors of the third type are cytosolic proteins. They are the NOD-like Receptors (NLRs) NOD1 and NOD2. Immune receptors perceive biological molecules. Surface receptors perceive extracellular molecules whereas cytosolic receptors perceive intracellular molecules. Both extracellular and intracellular molecules perceived by immune receptors can be either exogenous molecules, *i.e.*, not made by the organism, or endogenous molecules, *i.e.*, made by the organism. Examples of exogenous extracellular molecules are classical foreign antigens. Examples of endogenous extracellular molecules are autoantigens. Examples of exogenous intracellular molecules are microbial molecules produced by intracellular pathogens, such as bacterial peptidoglycans or viral nucleic acids. Likewise, although intracellular, vesicular receptors can perceive either exogenous extracellular molecules internalized from the outside, or endogenous intracellular molecules trafficking within the cell or transported from the inside to the outside.

As such, neither receptors expressed by sensory organs of the nervous system nor receptors expressed by cells of the immune system can distinguish between stimuli from the outer world and stimuli from the inner world. They can only signal that they have been engaged by stimuli that they can perceive. Due to their distribution in the body, the location of sensory receptors of the nervous system provides information on the origin of stimuli. However, thermoreceptors or baroreceptors respond similarly to variations of temperature and pressure, respectively, whether external or internal. Likewise, molecular patterns, nucleic acids, bacterial glycoproteins or other Microbe-Associated Molecular Patterns (MAMPs) perceived by TLRs, CLRs, NLRs or other PRRs are primarily the products of microorganisms, but these can be either outside pathogens or inside commensals. Besides, PRRs can also sense Damage-Associated Molecular Patterns (DAMPs) produced by altered cells in the body.

### Both the immune system and the nervous system undergo a selection process during their ontogeny.

The clonal selection theory proposed by Frank Macfarlane Burnet (31) accounts for the generation of the cognitive repertoire of the adaptive immune system. It rests on a unique genetic mechanism that was demonstrated to determine the antigen specificity of lymphocytes. Each lymphocyte (T or B) expresses antigen receptors (TCRs or BCRs) of a single specificity. The specificity of antigen receptors is determined by the association of one V gene, one D gene and one J gene, randomly chosen from three pools of V, D and J genes, respectively. This unique VDJ combination results from a somatic gene rearrangement that occurs in individual lymphocytes before they encounter antigen. When recognizing the antigen they are specific for under appropriate conditions, antigen receptors trigger signals that induce lymphocytes to proliferate and to form clones.

The somatic rearrangement being irreversible, rearranged VDJ genes are transmitted to daughter cells, and all lymphocytes in the clone that develops have the same specificity. Antigens therefore *select* lymphocytes that express preformed receptors and induce them to expand *clonally*. They also induce cell differentiation. T lymphocytes differentiate into effector T cells of various types (Th1, Th2, Th17 cells, cytotoxic T cells, regulatory T cells, etc.). Likewise, B lymphocytes differentiate into plasma cells that secrete antibodies with the specificity of the rearranged BCR, but also into effector and regulatory B cells (32). If antigens do not shape antibodies (or lymphocyte receptors), as claimed once by instructive theories (33), they shape the *repertoire* of the adaptive immune system by selecting an actual repertoire (resulting from antigen-driven clonal expansion) from an available repertoire (expressed by lymphocytes before they are selected), itself randomly sampled from a potential repertoire (made by all the possible combinations of V, D and J genes) (34).

It was on the model of Burnet’s clonal selection of lymphocytes that Gerald Edelman conceived the “selection of neuronal groups theory” (17). According to this theory, the brain cortex is organized in dynamic neuronal networks that are secondarily selected by experience. The nervous system develops early in fetal life under the control of the products of homeotic genes, and as a function of anatomical constraints. Neurons grow neurites and establish contacts with other neurons in the vicinity. Some start exchanging signals and survive. Others do not and die. The resulting self-generated system is highly diverse and varies between individuals of the same species. Later in infancy, information produced by experience and behavior generates extensive synaptic rearrangements. Synapses between already connected neurons and between groups of connected neurons are reinforced while less used synapses are eliminated. Experience thus selects strongly connected groups of neurons whose activities determine a secondary repertoire that further adapts to environmental constrains and stimuli. When fully mature in adults, the brain has gained a full capacity to respond specifically and/or to produce multiple solutions to perform a variety of tasks.

An experience-driven selection process therefore shapes both the adaptive immune system and the nervous system. Like lymphocytes generated with a randomly determined specificity, neurons first establish connections at random with close-by neurons. The available primary repertoires of lymphocytes and neurons are wide and diverse (one notices that the same term “repertoire” is used by immunologists to qualify the specificity of antigen receptors and by neurobiologists to qualify neuronal connections). An actual secondary repertoire is then selected by environmental factors. Like antigen determines the clonal expansion of lymphocytes whose receptors are engaged and the death of lymphocytes whose antigen receptors are not, experience determines the reinforcement of neuronal connections between groups of neurons involved in activity, and the elimination of connections that are not.

### Both the nervous system and the immune system use similar means of communication.

Soluble mediators used by the immune system have long been known to be also neurotransmitters. Classical examples are the monoamines histamine and serotonin. Histamine is stored in mast cell and basophil granules that are exocytosed upon IgE-induced cell activation. It accounts for itching in skin allergy, for increased vascular permeability associated with inflammatory reactions and, through the latter property, for blood pressure fall that can be fatal in anaphylaxis. Serotonin is stored in and, upon activation, is released by platelets. The largest source of serotonin, however, are enterochromaffin cells that modulate neuron signaling in the gut. Histamine and serotonin are also synthesized and released by histaminergic and serotonergic neurons, respectively. Both mediators are indeed neurotransmitters used by different neurons. Histaminergic neurons are mostly located in the hypothalamus, whereas serotoninergic neurons are disseminated in the central nervous system and the enteric nervous system. Both are involved in the sleep-wake cycle, but serotonergic pathways control a variety of other processes, including emotional behavior, circadian rhythm, appetite, and many visceral activities such as sexual behavior, and gastrointestinal movements (35). Likewise, the parasympathetic neurotransmitter acetylcholine was found to be produced by B lymphocytes and to negatively regulate hematopoiesis (36), by T lymphocytes and to control viral infection (37), and by type-2 Innate Lymphoid Cells (ILC2) and to promote anti-helminth immunity (38) (39).

For long, synapses designated contact zones between two neurons or between a motoneuron and a muscle cell, through which nervous impulse is transmitted. Transmission occurs when an action potential triggers the presynaptic neuron to release neurotransmitter-containing vesicles into the synaptic cleft that separates the two cells. When binding to neurotransmitter receptors expressed by the postsynaptic neuron or muscle cell, neurotransmitters trigger an action potential.

A synapse-like structure was first proposed to mediate cell-cell communication in the immune system in 1984. The analogy was soon adopted by immunologists, and synapses were redefined as “stable adhesive junctions between two cells across which information is relayed by directed secretion” (16). Immune synapses became a key structure accounting for antigen presentation to helper T cells (Th cells), cytotoxic T lymphocytes (CTL)-mediated and Natural Killer (NK) cell-mediated cytotoxicity (40), and in general, in direct cell-cell contacts involving immune cells.

Studies of synapses formed around TCR on Th cells and peptides bound to Major Histocompatibility Complex (MHC) class II molecules on dendritic cells (DCs), unveiled a tightly controlled molecular dynamics involving membrane molecules, intracellular signaling molecules and cytoskeleton molecules. These molecules form “Supramolecular activation clusters” (SMACs) (41), made by a *central SMAC* containing the TCR/MHC-peptide cluster, and by a *peripheral SMAC* containing the LFA-1 and ICAM-1 integrins, while microtubules are rearranged. *Src* family tyrosine kinases (Lck) are rapidly activated and cytosolic tyrosine kinases (ZAP-70) are recruited, and they initiate signals leading to T cell activation. Noticeably, the two cells involved in an immunological synapse, are successively presynaptic and post synaptic cells for the same synapse. In antigen presentation, DCs first deliver information to Th cells in form of class II MHC-peptide complexes, leading the T cell activation, and secondarily, activated Th cells deliver information to the same DCs in form of cytokines. Likewise, in cytotoxicity, target cells first deliver information to CTLs in form of class I MHC-peptide complexes, leading to T cell activation, and secondarily, activated CTLs deliver information to the same target cells in form of cytotoxic and proapoptotic molecules. In both cases, the synapse concentrates cytokines and cytotoxic mediators secreted by T cells in the synaptic cleft, and restricts their effects, which could otherwise affect a variety of cells, to the target cell involved in the synapse (42).

### Both the nervous system and the immune system have a memory

The idea that the immune system has a “memory” was tailored after that of the nervous system. Indeed, in common language, “memory” refers to the ability of the nervous system to “remember”. The analogy implies that the two systems can keep track of their previous experiences.

Neurological memory lies on variations of activity in neuronal networks. Memories depend on the activity of interconnected neurons in a particular space and time configuration. Sensorial experience indeed first enhances the efficacy of synapse transmission, especially synapses that use glutamate as a neurotransmitter (43). The new conformation of these synapses is then stabilized by the controlled expression and degradation of specific proteins (44). Neural memory therefore results from the long-term reorganization of neuronal networks whose activity is facilitated.

Immunological memory also depends on long-term changes in the immune system (45). Changes bear on lymphocyte populations. When confronted again to a given antigen, the immune system responds faster and more vigorously. Antibodies appear more rapidly in the blood, with higher titers and they persist longer. One reason is that an expansion of specific clones occurred during the first response, and they differentiated into effector cells and antibody-producing cells. Most of these cells disappear following the response. Some remain, however, a fraction of which, referred to as “memory lymphocytes” have a long life. A second stimulation therefore does not start from a naive but from a previously trained immune system.

Immune memory was long restricted to the adaptive immune system. Increasing evidence suggests that the idea of a trained immune system also applies to the innate immune system and, therefore, that innate immunity also has a memory (46). Unlike those of lymphocytes, immune receptors of myeloid cells and ILCs are germline encoded: they undergo no somatic gene rearrangement. Their memory depends on sustained, but reversible epigenetic changes induced by a previous stimulation, leading to an enhanced transcription of specific genes that were previously expressed upon activation. NK cell memory also results from their proliferation following stimulation by viruses. Trained innate immunity is increasingly thought to contribute to the long term “non-specific” effects of vaccines (47).

## Crosstalk between the two systems of relation

Analogies between biological objects or their ways of functioning are suggestive, but they tell us more about our way of seeing them than about objects themselves. They imply no objective link. Everyday increasing evidence, however, indicates that the nervous system and the immune system are not independent systems, but instead, that they function in concert.

### Microbes can affect nervous cells

Like immune cells, nervous cells can be activated by microbes and microbial products. Neurons are closely associated with myeloid cells, *i.e.*, glial cells, which, like innate myeloid cells of the immune system, can sense microorganisms and their soluble products *via* their PRRs. Neurons can also be activated by inflammatory mediators released or secreted by nearby immune cells, upon the engagement of their PRRs by microbes or microbial products, (see below).

Neurons can also be activated directly by microbial products. Thus, *Staphylococcus aureu*s can activate nociceptive sensory neurons *via* the release of bacterial N-formylated peptides and of the pore-forming toxin α-hemolysin. Activated nociceptors then release neuropeptides that modulate innate immune inflammation (48). Sulfolipid-1, a glycolipid produced by *Mycobacterium tuberculosis*, activates nociceptive neurons that induce cough in tuberculosis (49). Likewise, *Candida albicans* can directly activate sensory neurons and enhance host resistance to infection by secreting neuropeptides that drive the production of IL-23 by dendritic cells (50). Gut-innervating nociceptors can be activated by *Salmonella enterica* and release a neuropeptide that modulates both the density of Peyer patches’ M cells and the levels of segmentous filamented bacteria, leading to a protection against infection (51). A specific neuronal population of the central nucleus of the amygdala was found to be activated in the brain of mice recovering from experimental sepsis, leading to anxiety-related behavior and exaggerated fear memory similar to the long-term anxiety and post-traumatic stress disorder-like syndrome observed following sepsis in humans (52).

Finally, neurons themselves express PRRs (53), including TLRs and NLRs, which enable them to respond to microbial stimuli that engage these receptors. Inhibitory neurons of the hypothalamus can be activated by muropeptides produced by gut bacteria *via* the cytosolic PRR NOD2 and they regulate appetite and body temperature (54). LPS activates trigeminal neurons by sensitizing the ion channel TRPV1, when binding to TLR4 expressed by these neurons (55). Noticeably, TLRs signal through a unique mechanism in neurons. Instead of MYD88- TIRAP- or TRIF-dependent signaling pathways leading to the transcription of cytokines as in innate myeloid cells (56), TLRs expressed by nociceptive neurons are coupled to ion channels that rapidly modulate neuronal excitability, leading to the rapid onset of pain or itch (57).

Not surprisingly, the gut microbiota was found to profoundly affect the development and the functioning of the nervous system. While *in utero*, the fetus is exposed to metabolites produced by maternal gut microorganisms. It was shown in mice that these metabolites enable the establishment of proper connections between cortex and hypothalamus neurons, otherwise the offspring displays a variety of behavioral defects (58). The gut microbiota also induces the expression of a transcription factor in gastrointestinal neurons that control intestinal peristaltism in adult mice (59).

### Immune cells can affect nervous cells

When activated, immune cells can act on cells of the nervous system. As discussed above, immune cells can secrete acetylcholine, and several inflammatory mediators secreted by immune cells behave as neurotransmitters in the nervous system. Neurons also express cytokine receptors. Thus, the canonical pro-inflammatory cytokine IL-17 is produced by meningeal γδ T cells. There, it is not only involved in neuro-inflammatory diseases, it also activates cortical glutamatergic neurons that express the IL-17α receptor and modulates fear behavior in mice (60). Macrophages can both enhance pain by secreting proinflammatory mediators, and induce pain resolution by secreting anti-inflammatory mediators and endogenous opioids. Likewise, T cells promote both pain development following nerve injury, and pain resolution after transient inflammation (61). Type-2 cytokines such as IL-4 stimulate and activate sensory neurons that express IL-4Rα receptors and mediate chronic itch by enhancing neuronal responsiveness to pruritogens (62). IL-5, a cytokine involved in allergic airway inflammation, activates lung nociceptors that initiate cough and bronchoconstriction (63).

As expected, neurons are also the targets of effector cells of the immune system in neurodegenerative diseases. Tissue-resident inflammatory ILC3s can function as antigen-presenting cells and restimulate myelin-specific T cells in a murine model of multiple sclerosis (64). By killing neurons, NK cells determined the onset and progression of motor neuron degeneration in murine models of amyotrophic lateral sclerosis. By producing IFN-γ, NK cells can also induce an inflammatory phenotype in microglial cells, and impair the recruitment of FOXP3+/Treg cells in the CNS (65).

### Nervous cells can affect immune cells

Sensory neurons that express the ion channels TRPV1 and Nav1.8 were found to interact with and to induce dermal DCs to secrete IL-23 that drives skin inflammation in a murine model of psoriasis (66). Neuromedin U is a member of a family of neuropetides, with pleiotropic functions (67), especially in the gastrointestinal tract where it is produced by cholinergic neurons. It was recently found to trigger (68) and to amplify IL-25-induced (69) secretion of type-2 cytokines by ILC2s that express the NMUR1 receptor (70). Another neuropeptide, the vasoactive intestinal peptide (VIP), is secreted by the enteric nervous system in response to feeding. ILCs express high amounts of the VIP receptor VIPR2. VIP synergizes with IL-25 and IL-33, or with IL-1β and IL-23, to induce the production of high amounts of IL-5 by ILC2s or of IL-22 by ILC3s, respectively (71). ILC2s also express acetylcholine receptors and β2 adrenergic receptors, which control positively and negatively their functions, respectively. ILC2s themselves produce and secrete acetylcholine, and an exposure to helminths or allergens, which triggers the release of IL-25, IL-33 or TSLP, increases choline acetyltransferase, the enzyme responsible for acetylcholine synthesis, in ILC2s. Acetylcholine was found to induce ILC2 expansion, ILC2-dependent type-2 cytokine secretion and helminth expulsion (38) (39).

Conversely, β2 adrenergic receptor agonists such as norepinephrine were found to decrease type-2 responses by decreasing the proliferation and responses of ILC2s to helminth infection or allergen exposure (39) (72). Likewise, nociceptive sensory neurons were found to control herpes virus (HSV-1) infection in mice. They reduced skin inflammation by decreasing inflammatory cytokine secretion, monocyte activation and neutrophil infiltration in response to cutaneous infection by HSV-1, and by promoting CD8 T cell priming by skin DCs (73). Skin lymph nodes are indeed innervated by various subsets of sensory neurons, predominantly peptidergic nociceptors, whose stimulation by TLR agonists triggers rapid transcriptional changes in endothelium, stromal cells, and ILCs (74). Likewise, the lungs are innervated by sympathetic nerves, and norepinephrine or other agonists of β2-adrenergic receptors, negatively regulate LPS- or IL-33-elicited immune responses in the lung (75). Actually, most tissues including lymphoid organs are innervated by autonomic (primarily adrenergic) and sensory nerves. Both adrenaline, and substance P (produced by adrenergic neurons), or the neuropeptide TAFA4 (produced by sensory neurons) control leukocyte trafficking and the migration of immune cells in tissues [reviewed in (76)]. The above data and many others support and provide mechanistic explanations to the long known observation that stress may alter immune responses (77).

### Reciprocal crosstalk between the nervous system and the immune system

Altogether these two-way interactions between cells of the nervous and the immune system enable a multi-level reciprocal crosstalk between the two systems (78). I will underline two examples only: between neurons and ILC2s in allergy, and between the gut immune system and the insular cortex in inflammation.

ILC2s are key players in allergy, primarily as a source of type-2 cytokines. As discussed above, IL-4 activates sensory neurons that express IL-4 receptors (62), and activated neurons secrete neuromedin U that activates ILC2 that express neuromedin receptors (68) (70), thus generating an amplification loop that promotes allergic reactions (69). However, adrenergic neurons also secrete β2 adrenergic receptor agonists that inhibit the activation of ILC2 that express β2 adrenergic receptors, thus generating an inhibitory loop that dampens allergic reactions (72).

The second example bears on intestinal inflammation that can be induced by adding dextran sodium sulfate (DSS) in drinking water to generate a murine model of inflammatory bowel disease, with a massive infiltration of immune cells and gut wall leakiness. DSS-induced intestinal inflammation was found to activate distant neurons in the insular cortex. Following spontaneous recovery, gut inflammation could be re-induced in the absence of DSS, by stimulating insular neurons that were previously activated upon gut inflammation. Finally, a selective inhibition of insular neurons reduced gut inflammation induced by DSS. These experiments led to the identification of a novel neuronal pathway linking the intestine and the insula (79).

## The immune system unlike the nervous system

In spite of these similarities and functional interactions, the nervous and the immune system markedly differ. Among others, differences bear on their anatomical organization, on what they respond to and on what they act on.

### The two systems of relations markedly differ by their anatomy

Some systems, like the digestive system, the cardiovascular system or the nervous system, have a readily observed anatomical organization that strongly suggests their function. Others, like the hematopoietic system, the endocrine system or the immune system, lack such a suggestive organization.

The nervous system is indeed macroscopically “visible”: it can be dissected. Two large organs, the brain and the spinal cord, enclosed in bone containers, constitute the central nervous system (CNS). The CNS is prolonged by a network of sensory organs, nerves and ganglia, which constitute the peripheral nervous system (PNS). The PNS comprises the somatic nervous system and the autonomic nervous system, which innervate the body and internal organs, respectively. Unless connected by synapses, neural cells are separated from other cells. The immune system does not display such an obvious structure. It comprises lymphoid organs and vessels. Lymphoid organs comprise primary, secondary and tertiary organs. Primary lymphoid organs are the bone marrow and the thymus, in which immune cells differentiate from hematopoietic progenitors. Bone marrow is dispersed throughout the body in short and flat bones. The thymus regresses after birth. Secondary lymphoid organs comprise lymph nodes and the spleen in which adaptive immune responses are initiated and develop. Tertiary lymphoid organs are transient lymph node-like structures that form at inflammatory sites. Lymph nodes are connected by lymphatic vessels. Bone marrow and spleen are shared with the hematopoietic system. Lymphatic vessels merge with vessels of the vascular system. The immune system is an open “soft” system.

At the microscopic level, the nervous system is made by two main types of cells: neurons and glial cells. Neurons form both nuclei and nerves. Nuclei are made by neuronal bodies, while nerves are made by neuronal axons. Glial cells include microglial cells, astrocytes and oligodendrocytes in the CNS; Schwann cells in the PNS. Glial cells are myeloid cells associated with the nervous system. Immune cells also comprise two main types of cells: lymphoid cells and myeloid cells. Lymphoid cells comprise lymphocytes and ILCs. Classically, lymphocytes are primarily involved in adaptive immunity, whereas myeloid cells and ILCs are primarily involved in innate immunity. All three types of cells however collaborate in immune responses. Lymphocytes develop and differentiate in lymphoid organs, and circulate in the blood. ILCs are primarily tissue-resident cells. Myeloid cells circulate in the blood, home in tissues and/or shuttle between blood and tissues. Lymphoid and myeloid cells are therefore often mixed either with other blood cells in the circulation or with other cells in peripheral tissues.

In response to stimulation, the nervous system generates nervous impulses, *i.e.*, action potentials. Nervous impulses follow neuronal axons, jump from neuron to neuron *via* synapses and diffuse in neuronal networks. They travel *via* pre-established paths from the periphery to central organs and from central organs to the periphery. Signals triggered by immune receptors activate cells, leading to the production molecular and cellular effectors. Molecular effectors diffuse in the environment (*e.g.*, cytokines) and/or are carried by the blood throughout the body (*e.g.*, antibodies). They act on cells and molecules that bear specific targets (*eg.*, cytokine receptors or antigens). Cellular effectors follow the blood stream and/or migrate in tissues. Intermolecular contacts, molecule-cell contacts and intercellular contacts occur at random, as immune cells and molecules travel and encounter each other.

Altogether, these structural and functional differences make the immune system highly flexible, compared to the nervous system. Nervous responses triggered by given stimulus at a given time are largely predetermined, whereas immune responses are contingent and contextual.

### The two systems of relations have markedly different cognitive repertoires

The cognitive repertoires of the two systems can be defined as the whole spectrum of stimuli that can be perceived by nervous sensory receptors and by immune receptors, respectively.

The nervous system has the unique feature of being equipped with specific sensory organs that can perceive waves, vibrations, movements, pressure, temperature, etc., *i.e.*, physical manifestations. Rods and cones of the retina are stimulated by photons, *i.e.*, electromagnetic waves/particles; hairy cells of the internal ear by variations of fluid pressure generated by sound waves; aortic and carotid baroreceptors by blood pressure; Pacinian corpuscles of the skin by vibrations; Meissner’s corpuscles in the skin of finger pads by shapes and movements; Merckel discs of fingertips by the texture, shape and edges of physical objects; Ruffini corpuscles of the deep layer of the skin around fingernails to sustained pressure and mechanical deformation. Small molecules can also be perceived by taste and olfactory receptors through a variety of physicochemical properties. Salt receptors sense Na^+^ or NaCl; sour taste receptors free H^+^; bitter taste receptors cycloheximide, denatorium, propylthiouracil and β-glucopyranosides; sweet receptors sugars; umami receptors L-amino-acids. Olfactory receptors belong to a large multi-gene family. Each of them can perceive numerous volatile small-mw organic molecules, and each such molecule is perceived by numerous receptors.

As noted above, sensory neurons can be stimulated also by bacterial products *via* several mechanisms. One might therefore include microbial products in the cognitive repertoire of the nervous system. One notices, however, that many cells other than neurons can be similarly affected by a variety of molecules of microbial origin. TLRs were indeed found not only on nervous cells (57), including neurons, microglial cells, astrocytes (80) (81) and retinal photoreceptors (82), but also on epithelial cells in the gut (83) and the lung (84), on biliary and sinusoid epithelial cells, hepatocytes, Kupffer cells, stellate cells in the liver (85), on tubular epithelial cells and podocytes in kidneys, on urinary epithelial cells in the bladder (86), on cardiomyocytes (87), on endothelial cells in blood vessels (88), on myometrium and endometrium in the uterus (89), on amniotic, decidual and trophoblast cells (90), on β cells in the pancreas (91), as well as on ovarian (92), prostatic (93) and hematopoietic (94) cancer cells. PRR —especially TLR— expression is therefore a feature shared not only by immune and nervous cells, but also by a very large spectrum of non-immune, non-nervous cells, and sensing microbial products is not a unique property of the immune and the nervous system but a general property shared by of all these cells.

Waves, vibrations, pressure, temperature, stimuli perceived by most sensory receptors of the nervous system are molecular movements. Volatile substances and small molecules are perceived by olfactory and taste receptors through a limited number of specific properties. The cognitive repertoire of the nervous system consists in a limited number of *physicochemical phenomena*. This repertoire requires a small number of receptor types that have, each, a small repertoire. Many receptors, however can be engaged by a single stimulus. One volatile molecule, for instance, can bind to many, among the hundreds of different olfactory receptors present on the nasal epithelium. Signals generated by all these receptors are sent to the olfactory bulb, and processed in the amygdala, the orbitofrontal cortex and he thalamus. Although olfactory receptors have no specificity, the integration of multiple information emanating from multiple receptors enables one to discriminate smells very accurately. On the contrary, all sounds are sensed by one type of sound receptors only, with their differences in frequency, amplitude, and timbre. The external ear collects a limited spectrum of sound waves and it makes no difference between those that were produced by the violin and by the cello in a concert. Once transformed into nervous impulses by the hair cells in the organ of Corti, information is processed by the auditory cortex, and one can distinguish the parts played by different instruments of the orchestra, and integrate them in the symphony. A limited diversity of sensory receptors is sufficient to perceive stimuli from the physical world, but a big brain is needed to process and integrate them.

Immune cells are equipped not only with PRRs shared by nervous and other cells, but also with receptors that are not expressed by other cells, which can sense all living beings. These receptors perceive macromolecules. Macromolecules are specific of the living. These large molecules are long linear polymers with molecular weights ranging from thousands to millions of Daltons. They comprise essentially all proteins, including their variants (*e.g.*, glycoproteins, lipoproteins, etc.), but also nucleic acids under their different forms (*e.g.*, DNA, RNA, double-strain, single-strain, etc.). Macromolecules have a three-dimensional shape. The shape of nucleic acids is much simpler than that of proteins because polymers of four very similar nucleotides are much less polymorphic than polymers of twenty rather different amino-acids. In addition, proteins can be made of one or several polypeptide chains of variable lengths that fold into globular domains, due to the many physicochemical interactions between individual amino-acids in the same or in different polypeptides. Biological macromolecules bind to immunoreceptors that have complementary shapes. Binding indeed depends on multiple weak hydrophobic and electrostatic bonds between residues that are maintained close to each other in areas that have complementary shapes. Binding can involve more or less restricted areas of macromolecules. Some areas are formed by adjacent amino-acids in one polypeptide chain, some by distant sections of the same or of different polypeptides, which are brought close to each other by molecular folds. Others can belong to different molecules that are bound to each other, such as antigen-antibody complexes or MHC-peptide complexes on antigen-presenting cells. Some, borne by one or a few molecules only, bind to specific BCRs and antibodies; others, shared by numerous molecules borne by microorganisms, also bind to PRRs and NCRs.

Myriads of biological molecules can thus be perceived by the immune system. The cognitive repertoire of the immune system consists in a multitude of *biological objects*. Altogether, BCRs and corresponding antibodies can recognize more or less specifically an almost infinite number of proteins, TCRs a large number of MHC-peptide complexes, FcRs a multiplicity of antigen-antibody complexes. NCRs and PRRs can sense a variety of microbial lipoproteins, peptidoglycans, lipopolysaccharides, nucleic acids and proteins. A wide cognitive repertoire and a specific recognition imply the existence of a large number of immune receptors with a large diversity, expressed by a large number of immune cells of different types. A high diversity of immune receptors, but no central organ, is needed to perceive myriads of stimuli from the living world.

The cognitive repertoires of the two systems of relations therefore appear as belonging to two different worlds: phenomena of the physical world for the nervous system, objects of the living world for the immune system. This distinction being made, it raises the common-sense objection that the nervous system enables other living beings to be perceived, especially by human beings. It indeed enables them to see, to hear, to touch, to smell, to talk to each other. It is however not other living beings that sensory organs of the nervous system perceive, but their physicochemical manifestations: the light they reflect, the sound waves their vocal cords emit, the contact of their skin, the pressure of their fingers, the odors they release, etc. Supporting this statement, the nervous system enables even the perception of living beings in their absence. We can hear them talking on the radio or see them acting in a movie as if they were in front of us; we can read the books they wrote and listen to the music they composed long ago; we can see the trace they left once, in the silver grains of a photograph, long after they died. What the nervous system perceives are the physical manifestations of living beings, and these can be reproduced by machines.

### The two systems of relations have markedly different functional repertoires

The functional repertoires of the two systems can be defined as the spectrum of biological effects produced by effectors that can be generated and/or activated by the nervous system and by the immune system in response to the stimuli they perceive.

The functional repertoire of the nervous system is that of molecules secreted by neurons and that of cells and organs involved in responses to stimuli perceived by sensory organs. Neurons secrete a variety of neurotransmitters. These include amino acids, monoamines and peptides that are secreted in synaptic clefts where they mediate impulse transmission, but they are also secreted in extracellular medium where they function as local hormones. They are then referred to as neuromodulators. Neuromodulators primarily act on neuronal circuits (95), *i.e.*, on nervous cells. Some can also act on immune cells such as ILCs. Cellular effectors of the nervous system are muscles. The somatic nervous system primarily involves skeletal muscles *via* motoneurons. It controls both voluntary and reflex movements. The autonomous nervous system primarily involves smooth muscles located in organs. It affects the motility of the intestine and gallbladder, it closes and opens digestive and urinary sphincters, it modulates blood pressure, the rate and strength of contraction of the heart, it controls the diameter of bronchi, blood vessels, eye pupils, etc. The autonomous nervous system thus controls a variety of physiological phenomena. Altogether, responses of the nervous systems adapt the organism to physical changes perceived in the inner and the outer world. The nervous system induces behavioral responses, by acting on neurons and on muscles, *i.e.*, by acting on the organism.

The functional repertoire of the immune system is that of the molecular effectors secreted by immune cells and that of the many cellular effectors activated and/or recruited in response to the perception of biological macromolecules. Molecular effectors comprise cytokines and chemokines produced by immune cells when activated upon immune receptor engagement. Chemokines attract immune cells to reaction sites. Cytokines released in the extracellular medium activate surrounding cells that express corresponding receptors. These cells then perform biological activities enabled by their differentiation state. They include both immune cells and nervous cells. Molecular effectors also comprise the many antibodies secreted by plasma cells. Antibodies specifically bind to antigen-carrying cells or molecules *via* the variable regions of their two Fab portions, but they act on them *via* their Fc portions made of the constant 2-3 C-terminal domains of their heavy chains. A second somatic gene rearrangement indeed occurs in B cells, which brings the rearranged VDJ complex next to one heavy chain constant gene or another. For each antibody with a given specificity for antigen, nine classes and subclasses of antibodies with different Fc portions can thus be generated. Most if not all biological effects of antibodies depend on cells and molecules with which antibodies interact through their Fc portion. Molecules engaged by antibodies *via* their Fc portion are components of the complement system; cells are those that express FcRs (96).

Cellular effectors of the immune system include cells that differentiated from lymphocytes activated upon antigen receptor engagement, ILCs and myeloid cells. Activated naive T cells differentiate into Th1, Th2, Th17 cells that secrete various sets of cytokines, CTLs and regulatory T cells (Tregs) that act differentially on other cells. They also differentiate into memory T cells of several types. B cells primarily differentiate into antibody-secreting plasma cells, but also into memory or regulatory B cells. ILC1s, ILC2s, ILC3s secrete similar sets of cytokines as Th1, Th2 and Th17 cells, respectively, and NK cells have similar cytotoxic properties as CTLs. Following PRR or FcR engagement, the many and ubiquitous myeloid cells perform a variety of functions which depend primarily on the cell type. Altogether they contribute to internalization (endocytosis of soluble molecules or phagocytosis of cells and particles), antigen presentation, cytotoxicity and inflammatory reactions.

A major difference between the two systems of relations therefore bears on what they act on. Like effectors of the nervous system, effectors of the immune system can act on the organism (*i.e.*, immune cells and nervous cells), but unlike those of the nervous system, they act also on the *stimuli* that triggered a response. They can, for instance, kill a potential pathogen or keep it under control. The immune system can thus modify the living world the organism is confronted to. The nervous system cannot modify the physical world the organism lives in. Indeed, effectors of the nervous system act on muscles but not on stimuli perceived by sensory organs. Thus, for instance, when eyes are exposed to an intense light, the nervous system can induce the iris to reduce the pupils’ diameter, close eye lids and trigger a voluntary movement leading a human to put sunglasses on, but it has no means to act on the sun or the sun light. This distinction being made, one might argue that turning down the thermostat of a source of heat in response to the perception of a too high temperature in a room by skin thermoreceptors does affect the triggering stimulus. However, the response is not that of the nervous system itself but that of the hand and its muscles, *i.e.*, effectors that belong to other biological systems, in response to stimuli of the nervous system. Responses of the nervous system are ultimately those of the body, they are behavioral.

Another difference between the two systems of relations is the speed of their functioning. It takes a fraction of a second for a physical phenomenon to be perceived by cells of sensory organs and generate a nervous impulse, for this impulse to travel along axons, to go through synapses, to diffuse in neuronal networks, to be integrated in the SNC and to induce a muscle contraction. It takes hours for antigen to be processed and presented to the right lymphocytes. It takes days for lymphocytes to proliferate and differentiate into effector cells. It takes hours again for effector cells to find their targets, to be activated and to secrete effector molecules, and it takes additional minutes for these to act on the stimulus. Nervous responses that take less than one second are appropriate for physical stimuli that can occur rapidly and do not last long, such as lights and sounds for example. They enable the organism to interact with a fast-changing physical world. Immune responses that take days or weeks are well suited for biological stimuli that take time to establish and last long, such as microbial infections or the development of cancer cells. They enable the organism to adapt to and to act on an ever but slowly-changing living world.

## Conclusion

**Figure 1:**
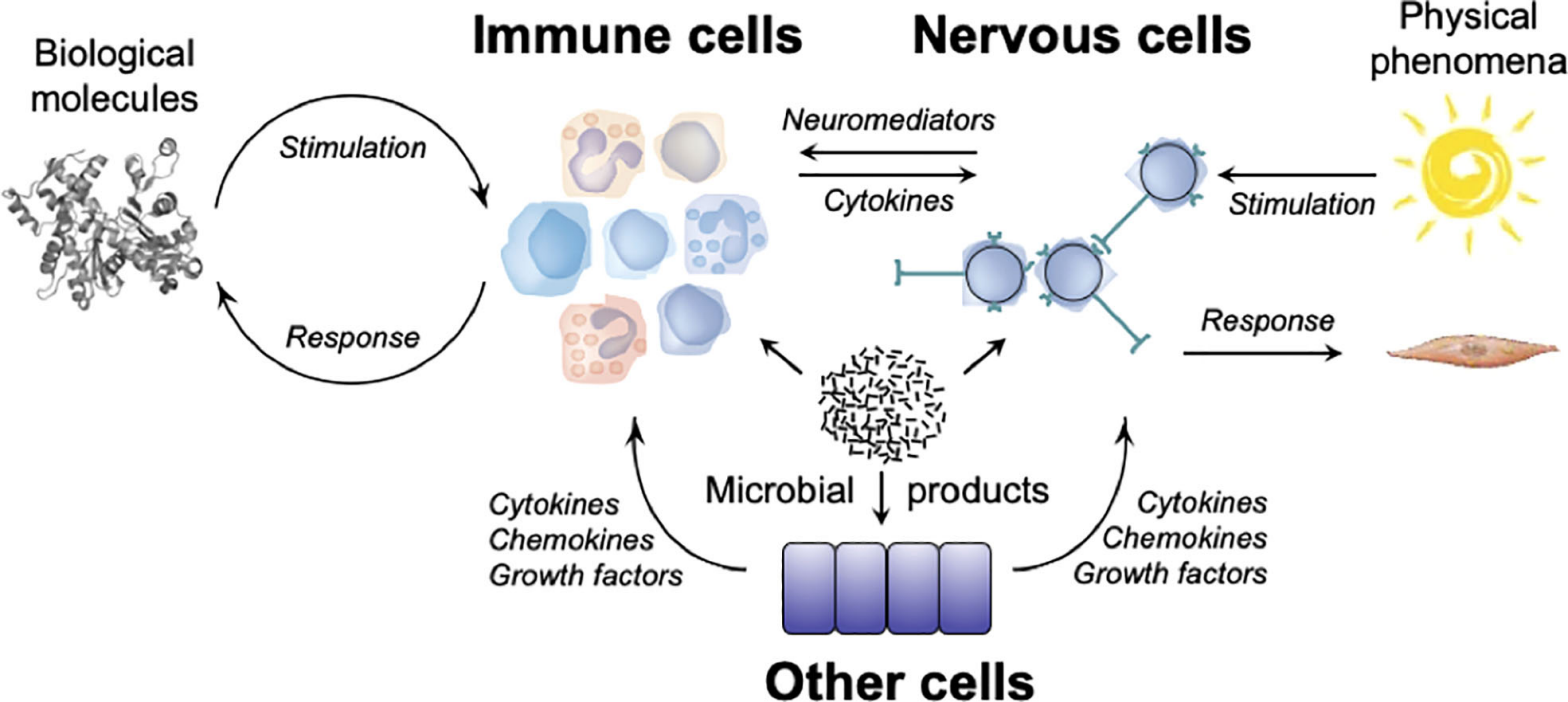
The cognitive and functional repertoires of the immune system and of the nervous system. Biological molecules stimulate immune cells that express immunoreceptors (TCRs, BCRs, NCRs) or pattern recognition receptors (TLRs, NLRs, CLRs), leading to the production and/or the activation of effector cells (T lymphocytes, ILCs, myeloid cells), and of molecules (cytokines, antibodies) which act on biological molecules that triggered the response. Physical phenomena stimulate sensory receptors of the nervous system which engender nervous influxes that propagate in neural networks, leading to the activation of effector cells (skeletal muscles, smooth muscles) that act on the organism. Microorganisms and/or their products can stimulate immune cells by engaging immunoreceptors (TCRs, BCRs, NCRs) on lymphocytes and ILCs, and pattern-recognition receptors (TLRs, NLRs) on myeloid cells, nervous cells (neurons and glial cells) and other cells (*e.g.* epithelial cells). Molecular and cellular immune effectors can act on organisms that triggered the response, but also on neurons that express cytokine receptors. Neuronal effectors can act on immune cells that express receptors for neuromediators. Effector molecules (*e.g.*, cytokines, chemokines, growth factors, etc.) secreted by other cells (*e.g.* epithelial cells) can act on immune cells (*e.g.*, ILCs) and nervous cells (*e.g.*, sensory neurons).

On the basis of the data reviewed above, one can understand the immune system as a system of relations rather than as a defense system. It cooperates with the nervous system, and the two systems control each other. The two systems, however have different cognitive and effector repertoires (**Figure 1**). As a result, the immune system has powerful adaptive properties that enable the organism to live in peace with itself and with other living beings, whether pathogens or commensals.

First of all, the immune system appears as a system of relations. Like the nervous system, it can perceive and respond not only to the outside world, but also to the inside world. This common property distinguishes systems of relations from other physiological systems. If they make possible the existence and the movements of the organism, systems of structure deal with the physical world. They enable the organism to live with physical constraints (*e.g.*, forces, gravity, etc.), but they do not perceive them. If they ensure the production, the degradation and the renewal of biological objects and living organisms, systems of maintenance deal with both the physical and the living worlds. They, however, have no means to sense them, and they do not respond to them. They internalize outside nutriments, gases, water, etc., sort them, transform and integrate some, degrade and excrete others, so that that the organism remains alive. The nervous and the immune systems enable the organism neither to use physical constraints nor to exploit outside resources. They enable it to sense the many stimuli of the world, whether the physical or the living world, whether from inside or from outside, whether harmful or harmless, and to respond to them.

Beyond analogies, the links are numerous between the two systems of relations. These links are direct and indirect. Like other organs, lymphoid organs, in which immune responses develop, are innervated and they respond to nervous stimuli. Cerebrospinal fluid was recently found to flow into the skull bone marrow and affect hematopoiesis in bacterial meningitis (97). Conversely, meningeal lymphatic vessels that carry immune cells and molecules irrigate the brain. As they use the same mediators/transmitters (*e.g.*, histamine, serotonin, acetylcholine, etc.), one system can be affected by soluble molecules produced by the other. Crosstalk takes place even when each system uses its own mediators, because neurons express receptors for effector molecules produced by immune cells (*e.g.*, cytokines), while immune cells express receptors for molecules produced by neurons (*e.g.*, neuropeptides). As a consequence, the nervous system and the immune system work in concert and they regulate, positively and negatively each other, in physiology, but also in pathology.

Marked differences, however, distinguish the immune system and the nervous system: they neither perceive nor act on the same things. Because they use different receptors, they have different cognitive repertoires: the nervous system can sense phenomena of the physical world, whereas the immune system can sense molecules of the living world. Because they share PRRs, the two systems can also sense microbes and microbial products and respond to them, as many other cells in the organism do, everywhere in the body. Because they use different molecular and cellular effectors, the immune and the nervous system have different functional repertoires. The nervous system acts on muscles, *i.e.*, on the organism but not on the stimulus that triggered the response. The immune system acts not only on immune and nervous cells, *i.e.*, on the organism, but also on the stimulus that generated the response. It has the double capability to adapt the organism to other living beings, and to adapt other living beings to the organism. Besides, the immune system has a greater flexibility than the nervous system. Its loose anatomical structure, the many molecular and cellular events that occur at random, the persistence of two large cognitive and functional potential repertoires that can be actualized at any time in any circumstance, confer to the immune system an exquisite ability to anticipate and to respond to the unexpected, including the unknown. For the best and for the worse. These unique adaptative properties, make it likely that the immune system has been, and still is, a major player in evolution.

Finally, the nervous system and the immune system appear as two complementary systems of relations that closely work together, and whose reactivities are suited for dealing with physical and biological stimuli, respectively. Whether they are two distinct physiological systems or two parts of a single system of relations may not be a critical issue as the answer depends on the ontologic value and the “granulosity” of functional units one uses to understand how living organisms live.

## Author contributions

The author confirms being the sole contributor of this work and has approved it for publication.

## Acknowledgments

I am grateful to Drs. Sophie Ugolini (Neural regulation of immunity, Centre d’immunologie de Marseille-Luminy, Marseille), Gérard Eberl (Microenvironment and Immunity, Institut Pasteur, Paris) and Pierre-Marie Lledo (Perception and Memory, Institut Pasteur, Paris) for their critical readings of the manuscript and their stimulating comments. This work was made possible thanks to an access to scientific literature provided to the author by the Institut Pasteur.

## Conflict of interest

The author declares that the research was conducted in the absence of any commercial or financial relationships that could be construed as a potential conflict of interest.

## Publisher’s note

All claims expressed in this article are solely those of the authors and do not necessarily represent those of their affiliated organizations, or those of the publisher, the editors and the reviewers. Any product that may be evaluated in this article, or claim that may be made by its manufacturer, is not guaranteed or endorsed by the publisher.

## Footnotes

1
https://www.physiology.org/career/teaching-learning-resources/student-resources/what-is-physiology?SSO=Y

2 The terms “to perceive” and “perception” will be used with a minimal meaning, as synonyms of “to sense” and “sensing”. They refer to the primary mechanism by which immune and nervous receptors generate intracellular signals in response to a stimulus. They do not imply the secondary processing of these signals that occurs in the central nervous system.

## References

[1] McGeer PL McGeer EG. The inflammatory response system of brain: Implications for therapy of Alzheimer and other neurodegenerative diseases. Brain Res Brain Res Rev (1995) 21(2):195–218. doi: 10.1016/0165-0173(95)00011-9 8866675

[2] Joshi N Singh S. Updates on immunity and inflammation in Parkinson disease pathology. J Neurosci Res (2018) 96(3):379–90. doi: 10.1002/jnr.24185 29072332

[3] Graus YMF De Baets MH. Myasthenia gravis: An autoimmune response against the acetylcholine receptor. Immunol Res (1993) 12(1):78–100. doi: 10.1007/BF02918370 7685805

[4] Wootla B Eriguchi M Rodriguez M. Is multiple sclerosis an autoimmune disease? Autoimmune Dis (2012) 2012:1–12. doi: 10.1155/2012/969657 PMC3361990 22666554

[5] Liao S Li C Bi X Guo H Qian Y Liu X. Anti-neuron antibody syndrome: clinical features, cytokines/chemokines and predictors. J Neuroinflammation (2021) 18(1):282. doi: 10.1186/s12974-021-02259-z 34872566 PMC8647466

[6] Vincent A Newsom Davis J. Anti-acetylcholine receptor antibodies. J Neurol Neurosurg Psychiatry (1980) 43(7):590–600. doi: 10.1136/jnnp.43.7.590 7400823 PMC490626

[7] Fletcher JM Lalor SJ Sweeney CM Tubridy N Mills KHG. T Cells in multiple sclerosis and experimental autoimmune encephalomyelitis. Clin Exp Immunol (2010) 162(1):1–11. doi: 10.1111/j.1365-2249.2010.04143.x 20682002 PMC2990924

[8] Smolders J Heutinck KM Fransen NL Remmerswaal EBM Hombrink P ten Berge IJM. Tissue-resident memory T cells populate the human brain. Nat Commun (2018) 9(1):4593. doi: 10.1038/s41467-018-07053-9 30389931 PMC6214977

[9] Anthony IC Crawford DH Bell JE. B lymphocytes in the normal brain: Contrasts with HIV-associated lymphoid infiltrates and lymphomas. Brain (2003) 126(5):1058–67. doi: 10.1093/brain/awg118 12690046

[10] Sriram S. Role of glial cells in innate immunity and their role in CNS demyelination. J Neuroimmunol (2011) 239(1–2):13–20. doi: 10.1016/j.jneuroim.2011.08.012 21907419

[11] Aspelund A Antila S Proulx ST Karlsen TV Karaman S Detmar M. A dural lymphatic vascular system that drains brain interstitial fluid and macromolecules. J Exp Med (2015) 212(7):991–9. doi: 10.1084/jem.20142290 PMC4493418 26077718

[12] Louveau A Smirnov I Keyes TJ Eccles JD Rouhani SJ Peske JD. Structural and functional features of central nervous system lymphatic vessels. Nature (2015) 523(7560):337–41. doi: 10.1038/nature14432 PMC4506234 26030524

[13] Ma Q Xing C Long W Wang HY Liu Q Wang RF. Impact of microbiota on central nervous system and neurological diseases: The gut-brain axis. J Neuroinflammation (2019) 16(1):53. doi: 10.1186/s12974-019-1434-3 30823925 PMC6397457

[14] Ansaldo E Farley TK Belkaid Y. Control of immunity by the microbiota. Annu Rev Immunol (2021) 39(1):449–79. doi: 10.1146/annurev-immunol-093019-112348 33902310

[15] Pradeu T Du Pasquier L. Immunological memory: What’s in a name? Immunol Rev (2018) 283(1):7–20. doi: 10.1111/imr.12652 29664563

[16] Dustin ML Colman DR. Neural and immunological synaptic relations. Science (2002) 298(5594):785–9. doi: 10.1126/science.1076386 12399580

[17] Edelman GM. Neural Darwinism: selection and reentrant signaling in higher brain function. Neuron (1993) 10(2):115–25. doi: 10.1016/0896-6273(93)90304-A 8094962

[18] Nutma E Willison H Martino G Amor S. Neuroimmunology - the past, present and future. Clin Exp Immunol (2019) 197(3):278–93. doi: 10.1111/cei.13279 PMC6693969 30768789

[19] Oltz EM. Neuroimmunology: To sense and protect. J Immunol (2020) 204(2):239–40. doi: 10.4049/jimmunol.1990024 31907263

[20] A neuroimmune odyssey. Nat Rev Immunol (2020) 20(4):203. doi: 10.1038/s41577-020-0293-6 32235933

[21] Blalock JE. The immune system as a sensory organ. J Immunol (1984) 132(3):1067–70. 6363533

[22] Kipnis J. Immune system: The “seventh sense”. J Exp Med (2018) 215(2):397–8. doi: 10.1084/jem.20172295 PMC5789422 29339443

[23] Cohen JA Edwards TN Liu AW Hirai T Jones MR Wu J. Cutaneous TRPV1+ neurons trigger protective innate type 17 anticipatory immunity. Cell (2019) 178(4):919–32.e14. doi: 10.1016/j.cell.2019.06.022 31353219 PMC6788801

[24] Trier AM Kim BS. Sensory neurons drive anticipatory immunity. Cell (2019) 178(4):771–3. doi: 10.1016/j.cell.2019.07.012 31398333

[25] Moulin AM. The immune system: A key concept for the history of immunology. History Philosophy Life Sci (1989) 11(2):221–36. 2700019

[26] Spoel SH Dong X. How do plants achieve immunity? Defence without specialized immune cells. Nat Rev Immunol (2012) 12(2):89–100. doi: 10.1038/nri3141 22273771

[27] Chen G Zhuchenko O Kuspa A. Immune-like phagocyte activity in the social amoeba. Science (2007) 317(5838):678–81. doi: 10.1126/science.1143991 PMC3291017 17673666

[28] Horvath P Barrangou R. CRISPR/Cas, the immune system of bacteria and archaea. Science (2010) 327(5962):167–70. doi: 10.1126/science.1179555 20056882

[29] Neander K. The teleological notion of ‘function.’ Australasian journal of philosophy (1991) 69(4):454–68. doi: 10.1080/00048409112344881

[30] Ratcliffe MJH. Encyclopedia of immunobiology (2016). Available at: http://lib.myilibrary.com?id=920225.

[31] Burnet FM. The clonal selection theory of acquired immunity. Nashville: Vanderbilt University Press (1959).

[32] Lund FE Randall TD. Effector and regulatory b cells: modulators of CD4+ T cell immunity. Nat Rev Immunol (2010) 10(4):236–47. doi: 10.1038/nri2729 PMC3038334 20224569

[33] Pauling LA. Theory of the structure and process of formation of antibodies. Linus pauling, “A theory of the structure and process of formation of antibodies,”. J Am Chem Soc (1940) 62:2643–57. doi: 10.1021/ja01867a018

[34] Coutinho A Kazatchkine MD Avrameas S. Natural autoantibodies. Curr Opin Immunol (1995) 7(6):812–8. doi: 10.1016/0952-7915(95)80053-0 8679125

[35] Berger M Gray JA Roth BL. The expanded biology of serotonin. Annu Rev Med (2009) 60(1):355–66. doi: 10.1146/annurev.med.60.042307.110802 PMC5864293 19630576

[36] Schloss MJ Hulsmans M Rohde D Lee IH Severe N Foy BH. B lymphocyte-derived acetylcholine limits steady-state and emergency hematopoiesis. Nat Immunol (2022) 23(4):605–18. doi: 10.1038/s41590-022-01165-7 PMC8989652 35352063

[37] Cox MA Duncan GS Lin GHY Steinberg BE Yu LX Brenner D. Choline acetyltransferase–expressing T cells are required to control chronic viral infection. Science (2019) 363(6427):639–44. doi: 10.1126/science.aau9072 PMC7181845 30733420

[38] Roberts LB Schnoeller C Berkachy R Darby M Pillaye J Oudhoff MJ. Acetylcholine production by group 2 innate lymphoid cells promotes mucosal immunity to helminths. Sci Immunol (2021) 6(57):eabd0359. doi: 10.1126/sciimmunol.abd0359 33674321

[39] Chu C Parkhurst CN Zhang W Zhou L Yano H Arifuzzaman M. The ChAT-acetylcholine pathway promotes group 2 innate lymphoid cell responses and anti-helminth immunity. Sci Immunol (2021) 6(57):eabe3218. doi: 10.1126/sciimmunol.abe3218 33674322 PMC8577047

[40] Davis DM Dustin ML. What is the importance of the immunological synapse? Trends Immunol (2004) 25(6):323–7. doi: 10.1016/j.it.2004.03.007 15145322

[41] Monks CRF Freiberg BA Kupfer H Sciaky N Kupfer A. Three-dimensional segregation of supramolecular activation clusters in T cells. Nature (1998) 395(6697):82–6. doi: 10.1038/25764 9738502

[42] Dustin ML. The immunological synapse. Cancer Immunol Res (2014) 2(11):1023–33. doi: 10.1158/2326-6066.CIR-14-0161 PMC4692051 25367977

[43] Kennedy MB. Synaptic signaling in learning and memory. Cold Spring Harb Perspect Biol (2016) 8(2):a016824. doi: 10.1101/cshperspect.a016824 PMC4743082 24379319

[44] Jarome TJ Helmstetter FJ. Protein degradation and protein synthesis in long-term memory formation. Front Mol Neurosci (2014) 7:61/abstract. doi: 10.3389/fnmol.2014.00061/abstract 25018696 PMC4072070

[45] Janeway CA Travers P Walport M Shlomchik M. Immunobiology. In: The immune system in health and disease, 5th edition. New York: Garland Science (2001).

[46] Netea MG Joosten LAB Latz E Mills KHG Natoli G Stunnenberg HG. Trained immunity: A program of innate immune memory in health and disease. Science (2016) 352(6284):aaf1098. doi: 10.1126/science.aaf1098 27102489 PMC5087274

[47] Blok BA Arts RJW van Crevel R Benn CS Netea MG. Trained innate immunity as underlying mechanism for the long-term, nonspecific effects of vaccines. J Leukocyte Biol (2015) 98(3):347–56. doi: 10.1189/jlb.5RI0315-096R 26150551

[48] Chiu IM Heesters BA Ghasemlou N Von Hehn CA Zhao F Tran J. Bacteria activate sensory neurons that modulate pain and inflammation. Nature (2013) 501(7465):52–7. doi: 10.1038/nature12479 PMC3773968 23965627

[49] Ruhl CR Pasko BL Khan HS Kindt LM Stamm CE Franco LH. Mycobacterium tuberculosis sulfolipid-1 activates nociceptive neurons and induces cough. Cell (2020) 181(2):293–305.e11. doi: 10.1016/j.cell.2020.02.026 32142653 PMC7102531

[50] Kashem SW Riedl MS Yao C Honda CN Vulchanova L Kaplan DH. Nociceptive sensory fibers drive interleukin-23 production from CD301b+ dermal dendritic cells and drive protective cutaneous immunity. Immunity (2015) 43(3):515–26. doi: 10.1016/j.immuni.2015.08.016 PMC4607048 26377898

[51] Lai NY Musser MA Pinho-Ribeiro FA Baral P Jacobson A Ma P. Gut-innervating nociceptor neurons regulate peyer’s patch microfold cells and SFB levels to mediate salmonella host defense. Cell (2020) 180(1):33–49.e22. doi: 10.1016/j.cell.2019.11.014 31813624 PMC6954329

[52] Bourhy L Mazeraud A Costa LHA Levy J Rei D Hecquet E. Silencing of amygdala circuits during sepsis prevents the development of anxiety-related behaviours. Brain (2022) 578:awab475. doi: 10.1093/brain/awab475 PMC9128826 35441215

[53] Liu T Gao YJ Ji RR. Emerging role of toll-like receptors in the control of pain and itch. Neurosci Bull (2012) 28(2):131–44. doi: 10.1007/s12264-012-1219-5 PMC3347759 22466124

[54] Gabanyi I Lepousez G Wheeler R Vieites-Prado A Nissant A Wagner S. Bacterial sensing via neuronal Nod2 regulates appetite and body temperature. Science (2022) 376(6590):eabj3986. doi: 10.1126/science.abj3986 35420957

[55] Diogenes A Ferraz CCR Akopian AN Henry MA Hargreaves KM. LPS sensitizes TRPV1 *via* activation of TLR4 in trigeminal sensory neurons. J Dent Res (2011) 90(6):759–64. doi: 10.1177/0022034511400225 21393555

[56] Takeda K Akira S. TLR signaling pathways. Semin Immunol (2004) 16(1):3–9. doi: 10.1016/j.smim.2003.10.003 14751757

[57] Donnelly CR Chen O Ji RR. How do sensory neurons sense danger signals? Trends Neurosci (2020) 43(10):822–38. doi: 10.1016/j.tins.2020.07.008 PMC7530006 32839001

[58] Vuong HE Pronovost GN Williams DW Coley EJL Siegler EL Qiu A. The maternal microbiome modulates fetal neurodevelopment in mice. Nature (2020) 586(7828):281–6. doi: 10.1038/s41586-020-2745-3 PMC7554197 32968276

[59] Obata Y Castaño Á Boeing S Bon-Frauches AC Fung C Fallesen T. Neuronal programming by microbiota regulates intestinal physiology. Nature (2020) 578(7794):284–9. doi: 10.1038/s41586-020-1975-8 32025031

[60] Alves de Lima K Rustenhoven J Da Mesquita S Wall M Salvador AF Smirnov I. Meningeal γδ T cells regulate anxiety-like behavior *via* IL-17a signaling in neurons. Nat Immunol (2020) 21(11):1421–9. doi: 10.1038/s41590-020-0776-4 PMC8496952 32929273

[61] Kavelaars A Heijnen CJ. Immune regulation of pain: Friend and foe. Sci Transl Med (2021) 13(619):eabj7152. doi: 10.1126/scitranslmed.abj7152 34757809 PMC8889025

[62] Oetjen LK Mack MR Feng J Whelan TM Niu H Guo CJ. Sensory neurons Co-opt classical immune signaling pathways to mediate chronic itch. Cell (2017) 171(1):217–28.e13. doi: 10.1016/j.cell.2017.08.006 28890086 PMC5658016

[63] Talbot S Abdulnour REE Burkett PR Lee S Cronin SJF Pascal MA. Silencing nociceptor neurons reduces allergic airway inflammation. Neuron (2015) 87(2):341–54. doi: 10.1016/j.neuron.2015.06.007 PMC4506220 26119026

[64] Grigg JB Shanmugavadivu A Regen T Parkhurst CN Ahmed A Joseph AM. Antigen-presenting innate lymphoid cells orchestrate neuroinflammation. Nature (202) 600(7890):707–12. doi: 10.1038/s41586-021-04136-4 PMC8702489 34853467

[65] Garofalo S Cocozza G Porzia A Inghilleri M Raspa M Scavizzi F. Natural killer cells modulate motor neuron-immune cell cross talk in models of amyotrophic lateral sclerosis. Nat Commun (2020) 11(1):1773. doi: 10.1038/s41467-020-15644-8 32286313 PMC7156729

[66] Riol-Blanco L Ordovas-Montanes J Perro M Naval E Thiriot A Alvarez D. Nociceptive sensory neurons drive interleukin-23-mediated psoriasiform skin inflammation. Nature (2014) 510(7503):157–61. doi: 10.1038/nature13199 PMC4127885 24759321

[67] Martinez VG O’Driscoll L. Neuromedin U: A multifunctional neuropeptide with pleiotropic roles. Clin Chem (2015) 61(3):471–82. doi: 10.1373/clinchem.2014.231753 25605682

[68] Klose CSN Mahlakõiv T Moeller JB Rankin LC Flamar AL Kabata H. The neuropeptide neuromedin U stimulates innate lymphoid cells and type 2 inflammation. Nature (2017) 549(7671):282–6. doi: 10.1038/nature23676 PMC6066372 28869965

[69] Wallrapp A Riesenfeld SJ Burkett PR Abdulnour REE Nyman J Dionne D. The neuropeptide NMU amplifies ILC2-driven allergic lung inflammation. Nature (2017) 549(7672):351–6. doi: 10.1038/nature24029 PMC5746044 28902842

[70] Cardoso V Chesné J Ribeiro H García-Cassani B Carvalho T Bouchery T. Neuronal regulation of type 2 innate lymphoid cells *via* neuromedin U. Nature (2017) 549(7671):277–81. doi: 10.1038/nature23469 PMC5714273 28869974

[71] Pascal M Kazakov A Chevalier G Dubrule L Deyrat J Dupin A. The neuropeptide VIP potentiates intestinal innate type 2 and type 3 immunity in response to feeding. Mucosal Immunol (2022) 15:629–41. doi: 10.1038/s41385-022-00516-9 35501356

[72] Moriyama S Brestoff JR Flamar AL Moeller JB Klose CSN Rankin LC. β _2_ -adrenergic receptor–mediated negative regulation of group 2 innate lymphoid cell responses. Science (2018) 359(6379):1056–61. doi: 10.1126/science.aan4829 29496881

[73] Filtjens J Roger A Quatrini L Wieduwild E Gouilly J Hoeffel G. Nociceptive sensory neurons promote CD8 T cell responses to HSV-1 infection. Nat Commun (2021) 12(1):2936. doi: 10.1038/s41467-021-22841-6 34006861 PMC8131384

[74] Huang S Ziegler CGK Austin J Mannoun N Vukovic M Ordovas-Montanes J. Lymph nodes are innervated by a unique population of sensory neurons with immunomodulatory potential. Cell (2021) 184(2):441–59.e25. doi: 10.1016/j.cell.2020.11.028 33333021 PMC9612289

[75] Liu T Yang L Han X Ding X Li J Yang J. Local sympathetic innervations modulate the lung innate immune responses. Sci Adv (2020) 6(20):eaay1497. doi: 10.1126/sciadv.aay1497 32426489 PMC7220323

[76] Mueller SN. Neural control of immune cell trafficking. J Exp Med (2022) 219(3):e20211604. doi: 10.1084/jem.20211604 35195682 PMC8932541

[77] Glaser R Kiecolt-Glaser JK. Stress-induced immune dysfunction: implications for health. Nat Rev Immunol (2005) 5(3):243–51. doi: 10.1038/nri1571 15738954

[78] Veiga-Fernandes H Artis D. Neuronal–immune system cross-talk in homeostasis. Science (2018) 359(6383):1465–6. doi: 10.1126/science.aap9598 29599230

[79] Koren T Yifa R Amer M Krot M Boshnak N Ben-Shaanan TL. Insular cortex neurons encode and retrieve specific immune responses. Cell (2021) 184(24):5902–15.e17. doi: 10.1016/j.cell.2021.10.013 34752731

[80] Song Y Shou LM Ai LY Bei Y Chen MT. Mini-review: The non-immune functions of toll-like receptors. Crit Rev Eukaryot Gene Expr (2019) 29(1):37–45. doi: 10.1615/CritRevEukaryotGeneExpr.2018027399 31002593

[81] Carty M Bowie AG. Evaluating the role of toll-like receptors in diseases of the central nervous system. Biochem Pharmacol (2011) 81(7):825–37. doi: 10.1016/j.bcp.2011.01.003 21241665

[82] Singh PK Kumar A. Retinal photoreceptor expresses toll-like receptors (TLRs) and elicits innate responses following TLR ligand and bacterial challenge. PloS One (2015) 10(3):e0119541. doi: 10.1371/journal.pone.0119541 25767877 PMC4358976

[83] Burgueño JF Abreu MT. Epithelial toll-like receptors and their role in gut homeostasis and disease. Nat Rev Gastroenterol Hepatol (2020) 17(5):263–78. doi: 10.1038/s41575-019-0261-4 32103203

[84] Ritter M Mennerich D Weith A Seither P. Characterization of toll-like receptors in primary lung epithelial cells: strong impact of the TLR3 ligand poly(I:C) on the regulation of toll-like receptors, adaptor proteins and inflammatory response. J Inflammation (Lond) (2005) 2:16. doi: 10.1186/1476-9255-2-16 PMC1315317 16316467

[85] Seki E Brenner DA. Toll-like receptors and adaptor molecules in liver disease: update. Hepatology (2008) 48(1):322–35. doi: 10.1002/hep.22306 18506843

[86] Anders HJ. Innate pathogen recognition in the kidney: Toll-like receptors, NOD-like receptors, and RIG-like helicases. Kidney Int (2007) 72(9):1051–6. doi: 10.1038/sj.ki.5002436 17653134

[87] Shintani Y Drexler HCA Kioka H Terracciano CMN Coppen SR Imamura H. Toll-like receptor 9 protects non-immune cells from stress by modulating mitochondrial ATP synthesis through the inhibition of SERCA2. EMBO Rep (2014) 15(4):438–45. doi: 10.1002/embr.201337945 PMC3989675 24610369

[88] Wang Y Song E Bai B Vanhoutte PM. Toll-like receptors mediating vascular malfunction: Lessons from receptor subtypes. Pharmacol Ther (2016) 158:91–100. doi: 10.1016/j.pharmthera.2015.12.005 26702901

[89] Gonzalez JM Xu H Ofori E Elovitz MA. Toll-like receptors in the uterus, cervix, and placenta: is pregnancy an immunosuppressed state? Am J Obstetrics Gynecol (2007) 197(3):296.e1–6. doi: 10.1016/j.ajog.2007.06.021 17826427

[90] Koga K Mor G. Toll-like receptors at the maternal-fetal interface in normal pregnancy and pregnancy disorders: TLRS AND PREGNANCY. Am J Reprod Immunol (2010) 63(6):587–600. doi: 10.1111/j.1600-0897.2010.00848.x 20367625 PMC3025804

[91] Garay-Malpartida HM Mourão RF Mantovani M Santos IA Sogayar MC Goldberg AC. Toll-like receptor 4 (TLR4) expression in human and murine pancreatic beta-cells affects cell viability and insulin homeostasis. BMC Immunol (2011) 12(1):18. doi: 10.1186/1471-2172-12-18 21356084 PMC3060152

[92] Kelly MG Alvero AB Chen R Silasi DA Abrahams VM Chan S. TLR-4 signaling promotes tumor growth and paclitaxel chemoresistance in ovarian cancer. Cancer Res (2006) 66(7):3859–68. doi: 10.1158/0008-5472.CAN-05-3948 16585214

[93] Rezania S Amirmozaffari N Rashidi N Mirzadegan E Zarei S Ghasemi J. The same and not the same: heterogeneous functional activation of prostate tumor cells by TLR ligation. Cancer Cell Int (2014) 14:54. doi: 10.1186/1475-2867-14-54 24966802 PMC4069277

[94] Monlish DA Bhatt ST Schuettpelz LG. The role of toll-like receptors in hematopoietic malignancies. Front Immunol (2016) 7:390. doi: 10.3389/fimmu.2016.00390 27733853 PMC5039188

[95] Marder E. Neuromodulation of neuronal circuits: Back to the future. Neuron (2012) 76(1):1–11. doi: 10.1016/j.neuron.2012.09.010 23040802 PMC3482119

[96] Daëron M. Fc receptors as adaptive immunoreceptors Vol. 382. Daeron M Nimmerjahn F, editors. Cham: Springer International Publishing (2014) p. 131–64. Available at: http://link.springer.com/10.1007/978-3-319-07911-0_7 10.1007/978-3-319-07911-0_7 PMC7120570 25116099

[97] Pulous FE Cruz-Hernández JC Yang C Kaya Z Paccalet A Wojtkiewicz G. Cerebrospinal fluid can exit into the skull bone marrow and instruct cranial hematopoiesis in mice with bacterial meningitis. Nat Neurosci (2022) 25(5):567–76. doi: 10.1038/s41593-022-01060-2 PMC9081225 35501382

