# Supplementary material for: The immune system as a system of relations
Source: Front Immunol. 2022 Sep 13;13:984678. doi: 10.3389/fimmu.2022.984678 (PMC9513551; doi:10.3389/fimmu.2022.984678)
Supplement: Supplementary file 1 [file Table_1.pdf]

**Table 1**: Common features of the immune and the nervous systems

| The immune system | The nervous system |
|---|---|
| ***A system of relations with the outer and the inner worlds*** | |
| Membrane (immunoreceptors, PRRs) and cytosoloic (NLRs) immune receptors | Head and skin sensory organs and visceral sensory receptors |
| ***Ontogenic development through a selection process*** | |
| Random selection of an available cognitive repertoire | Random selection of a primary neural network |
| Ag-driven selection of an actual cognitive repertoire | Experience-driven selection of an actual neural network |
| ***Response to Microbes*** | |
| Microbial products activate lymphoid and myeloid cells | Microbial products activate nociceptive sensory receptors |
| ***Intercellular communication*** | |
| Immune mediators (histamine, serotonin, etc.) | Neurotransmitters (histamine, serotonin, etc.) |
| Immunological synapses | Neuronal synapses |
| ***Memory of past experience*** | |
| Long-term changes in lymphocyte populations | Long-term changes in neuronal networks |

**Table 2**: Diffferences between the immune and the nervous systems

| The immune system | The nervous system |
|---|---|
| ***Conditions of cellular and molecular interactions*** | |
| Loose anatomical structure | Clear anatomical structure |
| Shared transport means (blood vessels) | Proper transport means (nerves) |
| Mixture of immune cells with blood and and tissular cells | Specific location of neurons in nervous organs |
| Non-predetermined synapse formation | Largely predetermined synapse formation |
| ***Cognitive repertoires*** | |
| Macromolecules of the biological world | Physicochemical manifestations of the physical world |
| Large number of specific receptors | Small number of poorly specific receptors |
| No integration of immune signals in central organ(s) | Integration of nervous signals in large central organs |
| Large potential repertoire that can be actualized at any time | An actual repertoire, but no potential repertoire |
| ***Effector repertoires*** | |
| Effector cells and molecules that act on biological molecules that triggered the response | Neurotransmitters that act on skeletal and visceral muscles leading to a behavioral response |
| ***Kinetics of responses*** | |
| Slow responses (days) | Fast responses (fraction of a second) |
